# Asthma control and opportunities to optimize management and the healthcare provider experience using the AsthmaOptimiser online tool in Dutch general practice: the CAPTURE study

**DOI:** 10.1038/s41533-025-00427-9

**Published:** 2025-04-15

**Authors:** Marika T. Leving, Yoran H. Gerritsma, David J. Jackson, Erik W. M. A. Bischoff, Jiska M. Meijer, Hans Wouters, Bertine Flokstra-de Blok, Janwillem W. H. Kocks

**Affiliations:** 1 General Practitioners Research Institute, Groningen, The Netherlands; 2 Guy’s Severe Asthma Centre, Guy’s & St Thomas’ NHS Trust, London, United Kingdom; 3 School of Immunology & Microbial Sciences, King’s College London, London, United Kingdom; 4 Department of Primary and Community Care, Radboud Institute for Health Sciences, Radboud University Medical Centre, Nijmegen, The Netherlands; 5 University of Groningen, University Medical Center Groningen, GRIAC Research Institute, Groningen, The Netherlands; 6 University of Groningen, University Medical Center Groningen, Beatrix Children’s Hospital, Department of Pediatric Pulmonology and Pediatric Allergology, Groningen, The Netherlands; 7 Observational and Pragmatic Research Institute, Singapore, Singapore; 8 Department of Pulmonology, University of Groningen, University Medical Center Groningen, Groningen, The Netherlands

**Keywords:** Outcomes research, Asthma

## Abstract

Patients seen in general practices can achieve improved asthma control with better identification of factors that contribute to uncontrolled asthma. Information is lacking on the proportion of patients with uncontrolled asthma, associated patient characteristics, and opportunities to improve management. The objectives of this study were to determine the proportion of general practice patients with uncontrolled asthma, as assessed during a regular consultation with the AsthmaOptimiser digital tool, identify the opportunities for improved management, and to evaluate the usability of this tool which is based on treatment recommendations from GINA. The CAPTURE study was a non-interventional, prospective, observational study of the AsthmaOptimiser in general practice settings in the Netherlands. Patients were at least 18 years of age with an asthma diagnosis. A total of 34 Dutch general practitioners or nurse practitioners participated in the study and planned to use the AsthmaOptimiser with 5 to 10 adult patients per practice. Interviews were conducted to gather information from practitioners about the tool’s usability, its content, and areas for improvement. Of the 220 patients enrolled, 60% had uncontrolled asthma, of whom 64% had opportunities for management improvement that could be initiated during a primary care visit. Specialist referrals were advisable according to the AsthmaOptimiser in 45 patients with uncontrolled asthma. Practitioners reported that the AsthmaOptimiser was an added value and had suggestions on how to improve the tool. In Dutch general practices, the AsthmaOptimiser helped general practitioners identify opportunities for improved disease management by addressing poor disease control. Overall, the general practitioners found the AsthmaOptimiser easy to use and a good addition to asthma consultations.

## Introduction

Asthma is a heterogeneous respiratory disease that is frequently associated with airway hyperresponsiveness, e.g., wheezing, shortness of breath, chest tightness, and chronic airway inflammation^1^. Asthma affects more than 339 million adults and children worldwide^2^, and approximately 5% to 10% of patients with asthma have severe asthma^3^. Severe asthma is associated with oral corticosteroid use, reduced health-related quality of life, increased disease burden (e.g., limits on activities of daily living, functional independence, work, and relationships), morbidity (e.g., complications of oral corticosteroid use such as sleep disorders, anxiety, depression, and hypertension), and economic burden^4–6^.

Patients with controlled asthma have a lower use of healthcare resources, fewer exacerbations, and the potential to reduce their use of asthma medication^1^. By contrast, patients with uncontrolled asthma are at risk for acute exacerbations that may require treatment with corticosteroids and other medications, which may lead to hospitalisation. Acute exacerbations requiring treatment can be associated with adverse events from oral corticosteroid use, increased utilisation of healthcare resources, poor health-related quality of life, and, in some cases, death^1,7–10^. Not all patients with uncontrolled asthma have severe asthma^1,10,11^.

Modifiable factors that can contribute to uncontrolled asthma include poor medication adherence, incorrect inhaler technique, and unmanaged comorbidities (e.g., obstructive sleep apnoea, rhinosinusitis, obesity, and gastroesophageal reflux disease)^1,11,12^. In 1 study, 56% of patients identified as having difficult-to-treat asthma were classified as such because of either poor adherence or incorrect inhaler technique^11^. Most patients with asthma are treated in general practice. However, there may be instances when a referral to a specialist can offer better treatment options for patients with persistent symptoms and help address factors that contribute to poor disease control^12^. Failure to refer patients to specialists can contribute to worse outcomes and possibly lead to avoidable deaths^12,13^. A national investigation of deaths attributed to asthma in the United Kingdom between 2012 and 2013 found that 57% of patients with asthma who died had no record of being under specialist care during the 12 months prior to death^13^. In 43% of patients with asthma who died, there was no evidence of an asthma review in general practice in the year preceding their death^13^. Deaths occurred in patients with a range of asthma severities (i.e., mild, moderate, and severe)^13^.

Digital asthma assessment tools have been developed for use by healthcare professionals to identify patients with potentially severe or uncontrolled asthma who require additional support to optimise disease management^14^. Structured questions in these tools can support comprehensive asthma assessments by providing healthcare professionals with personalised, guidelines-based strategies to optimise asthma management. ReferID^+^ is one such digital tool that has demonstrated significant reductions in exacerbations and improvements in asthma control compared to standard care in an 18-month study in the United Kingdom (the OASIS study [NCT04941001])^15–17^. This tool consists of a panel of asthma assessment questions designed to guide structured consultations to ensure critical elements are addressed and asthma treatments are optimised.

AsthmaOptimiser, a digital interface tool adapted from the ReferID^+^ tool specifically for the Dutch healthcare system, is intended for use by healthcare professionals in the Netherlands to conduct structured asthma assessments, which can help identify and address the specific needs of their patients. The AsthmaOptimiser tool creates an overview of factors and characteristics that may be associated with uncontrolled asthma based on GINA recommendation as well as guidelines-based treatment recommendations. The adaptation of the tool into the Dutch language allowed for the inclusion of additional asthma control questionnaires (e.g., Asthma Control Questionnaire, 6 items [ACQ-6]) and electronic medical record system integration.

The objectives of the CAPTURE study were to determine the proportion of general practice patients with uncontrolled asthma, as assessed during a regular consultation with the AsthmaOptimiser digital tool, to identify opportunities for improvements in disease management^18^. The study also sought to identify associations between levels of asthma control and patient demographics, work productivity and activity impairment (WPAI) assessment scores^19^, and blood eosinophil (bEOS) counts. Finally, the study captured user feedback on the AsthmaOptimiser, specifically related to the ease of use, points of improvement, and when and why the AsthmaOptimiser treatment recommendations were or were not subsequently implemented with patients.

## Methods

### Study design

The CAPTURE study (NCT04456270) was a non-interventional, prospective, observational study in general practice settings in the Netherlands. Care was delivered as usual, except for the use of the AsthmaOptimiser as part of the consultation. A second, qualitative part of the study involved surveys and face-to-face interviews, which were used to collect data on the AsthmaOptimiser’s usability and content and suggestions for improvement from the healthcare providers who participated in the study.

### Participants

This study involved general practice patients aged 18 years or older with a current asthma diagnosis who attended a regular general practice asthma review in the Netherlands. The participation of each enrolled patient was limited to 1 visit. Patients with a life expectancy of less than 1 year, who did not understand Dutch, or who had any other conditions believed to present a safety risk or that might impact the study results were excluded. The study was performed by general practice nurses (GPNs), who provide most of the routine asthma care in Dutch general practices. We aimed to include 30 Dutch general practitioners and GPNs, and we proposed that each healthcare practitioner use the AsthmaOptimiser with 5 to 10 adult patients.

### Study procedures

#### Quantitative procedures

Patients received standard medical care as determined by their physician. Two extra elements were added to the visit: a WPAI questionnaire^19^ and optional point of care measurement of blood eosinophils using a fingerpick test. Prior to the consultation, patients were asked to fill in the WPAI questionnaire. The WPAI questionnaire provides self-reported information on absenteeism (i.e., work time missed), presenteeism (i.e., impairment at work/reduced on-the-job effectiveness), and impairment of activities due to overall health and symptoms^19^.

During the patient consultation, the nurse provided the usual standard of care by collecting information on patient characteristics and asthma-related factors electronically into the AsthmaOptimiser. Any additional information that was needed after the visit was accessed through electronic records of the general practices. We evaluated the recommendation for referral to a specialist from the AsthmaOptimiser to identify how many patients had a referral and how many were scheduled. We also performed follow-ups with the GPNs to understand what factors were considered in their decision-making to schedule or not schedule a referral to a specialist and the reasons patients were not scheduled for a referral.

#### The AsthmaOptimiser

The AsthmaOptimiser is available via a secure website (www.asthmaoptimiser.com) which – after completing a login – presents the steps to perform for a structured asthma consultation, based on recommendations from GINA. For example, these steps include assessment of exacerbation history, attitudes towards medication use, calculation of adherence level, asthma triggers and comorbidities, and finally videos about, and assessments of, inhaler technique. The ACQ-6 is used for the assessment of asthma control, and the number of exacerbations are assessed via the following questions: 1) how many prescriptions of systemic corticosteroids has the patient received for asthma over the past 12 months, and 2) how many times has the patient had an emergency attendance, admission, or unscheduled visit due to asthma over the past 12 months (never; 1 time; 2 or more times). Based on the provided responses and decision trees within the AsthmaOptimiser tool, the healthcare provider receives GINA-based treatment recommendations and has the option to transfer all of the recorded data into the electronic medical record and/or generate a referral letter including all information. Example screenshots for hypothetical responses to AsthmaOptimiser questions for a patient in the CAPTURE study are shown in the Supplemental Appendix.

#### User feedback procedures

We conducted individual interviews with the GPNs, either in person or through video calls. All participating GPNs were interviewed twice using an interview guide: (1) before they started using the AsthmaOptimiser to learn about their expectations and (2) after they had used the AsthmaOptimiser to learn about their experience. Interviews were semi-structured and were recorded with permission from the respondent and subsequently transcribed to text. GPNs provided feedback about their expectations and perspectives on the AsthmaOptimiser and decisions they made for patient care using information obtained from the tool (e.g., considerations for not following the advice to refer the patient to a specialist and whether a referral was scheduled in the future). Surveys were sent to the healthcare providers asking for demographic data and for them to rate, by importance, suggestions for improvements to the AsthmaOptimiser tool.

### Data analysis

#### Quantitative statistical analyses

Descriptive statistics were used to summarise patient background, management suggestions, and WPAI results, using means with SDs for interval variables and percentages for nominal variables.

The primary objective was to identify the proportion of people with uncontrolled asthma. Secondary objectives were examined with generalised linear mixed models using the logit-link function using the lme4 package (v1.1-21)^20^. Asthma control (controlled or uncontrolled) was used as the dependent variable, and demographic characteristics, WPAI assessment scores, or bEOS counts were included as independent variables. Each model included a random intercept at the level of general practices to account for potential dependence of observations (i.e., patients from the same general practice). From these models, we calculated the mean (SD) for WPAI assessment scores and bEOS counts in patients with controlled and uncontrolled asthma and estimated odds ratios (ORs) with 95% CIs.

Missing values were imputed by using multiple imputation, and each missing value was replaced with an average of 3 independent imputations. Sensitivity analyses were conducted, e.g., complete-case analysis excluding imputed data. If models for complete-case analysis and imputed data provided similar results (i.e., overlap of 95% CI with 1), we reported the OR and 95% CI for the complete-case analysis only. Analyses were performed in R v.3.5.3^21^ in the Rstudio IDE v.1.3.1073 (Posit Software, Boston, MA, USA)^22^.

#### User feedback analysis

The demographic data from the survey results were analysed. The feedback received from the interviews was reviewed to identify emerging patterns and themes. An inductive thematic analysis of the interviews was performed, and the quotes from the respondents were assigned to these themes. Data saturation was reached within the recorded interviews.

### Ethics

This trial was conducted in accordance with the principles of the Declaration of Helsinki. Prior to study initiation, the clinical study protocol was reviewed and declared low-risk (non-WMO), by the medical research ethics committee “Stichting Beoordeling Ethiek Biomedish Onderzoek (BEBO)”. The study is registered on clinicaltrials.gov, NCT04456270. All patients provided written informed consent prior to enrolment.

## Results

**Fig. 1:**
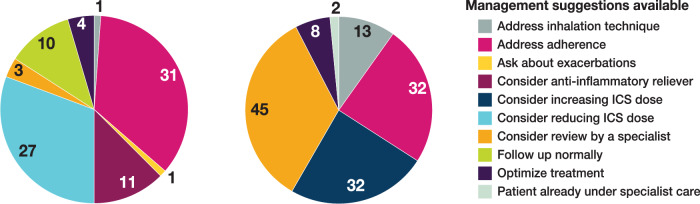
Distribution of guideline-based management suggestions provided by the AsthmaOptimiser tool for patients with controlled versus uncontrolled asthma. Total population = 220. Left: patients with controlled asthma (n = 88, 40%). Right: patients with uncontrolled asthma (n = 132, 60%). ICS inhaled corticosteroids.

There were 220 patients enrolled in the study; on average, each practice included 7.93 patients (minimum: 0, maximum: 36). Overall, women accounted for 63.6% of patients, and the patients’ mean (SD) age was 52.1 (17.9) years (Table 1). In total, 132 patients (60%) had uncontrolled asthma on the basis of an ACQ score of 0.75 or higher (Fig. 1). Women were at greater odds of having uncontrolled asthma (OR, 1.88; 95% CI, 1.01–3.50; *p* = 0.046), and odds of having uncontrolled asthma decreased with age (OR, 0.98; 95% CI, 0.96–0.99; *p* = 0.009). Individuals with lower socioeconomic status were at greater odds of having uncontrolled asthma (OR, 2.65; 95% CI, 1.09–6.44; *p* = 0.031).

**Table 1:** Descriptive statistics of patients in the CAPTURE study.

Characteristic	Controlled asthma (n = 88)	Uncontrolled asthma (n = 132)	Overall (N = 220)
**Sex, No. (%)**			
Male	37 (42.0)	39 (29.5)	76 (34.5)
Female	49 (55.7)	91 (68.9)	140 (63.6)
Missing	2 (2.3)	2 (1.5)	4 (1.8)
**Age, y**			
Mean (SD)	55.9 (16.7)	49.6 (18.2)	52.1 (17.9)
Median (min, max)	57.5 (20.0, 87.0)	51.0 (20.0, 85.0)	54.0 (20.0, 87.0)
**Ethnicity, No. (%)**			
White	20 (22.7)	24 (18.2)	44 (20.0)
African American	1 (1.1)	0 (0)	1 (0.45)
Missing	67 (76.1)	108 (81.8)	175 (79.5)
**Working status, No. (%)**			
Employed	51 (58.0)	79 (59.8)	130 (59.1)
Unemployed	34 (38.6)	51 (38.6)	85 (38.6)
Missing	3 (3.4)	2 (1.5)	5 (2.3)
**Socioeconomic limitations present,**^**a**^ **No. (%)**			
Yes	13 (14.8)	25 (18.9)	38 (17.3)
No	59 (67.0)	90 (68.2)	149 (67.7)
Missing	16 (18.2)	17 (7.7)	33 (15.0)

*Min* minimum, *Max* maximum.

^a^Socioeconomic limitations present included questions about financial status, living situation (e.g., mold or dust), work-related limitations, education level, and others. The table represents how many individuals had ≥1 limitation present.

Of the 132 patients with uncontrolled asthma, 85 patients (64%) had modifiable factors that could be addressed during the consultation to optimise the patient’s asthma control (e.g., addressing inhaler adherence or considering an increase in the patient’s inhaled corticosteroid dose). Referrals to a specialist were advised for 45 patients with uncontrolled asthma, of whom 2 patients were already receiving specialist care (Fig. 1). The 2 most frequently reported reasons for referral recommendations occurred in patients with uncontrolled asthma and were adherence issues (n = 25 [58.1%]) and worsening of asthma symptoms at work (n = 25 [58.1%], Table 2). Only 8 of 45 patients with uncontrolled asthma identified for a referral were eventually referred after the consultation in which the AsthmaOptimiser was used, often after having an additional consultation. These 8 patient referrals were made because of persistent shortness of breath, decreased lung function, symptoms after a change in medication, adverse effects from medications, lung attack or pneumonia, nasal polyps, persistent symptoms, and the results of a sleep apnoea test (in 2 patients). Reasons for non-referral are described in Supplement S1.

**Table 2:** CAPTURE study underlying reasons for advice to schedule a specialist referral.

Underlying reason for referral, No. (%)	Controlled asthma (n = 3)	Uncontrolled asthma (n = 45)
Adherence <80%	3 (100)	25 (58.1)
Asthma symptoms worse at work	2 (66.6)	25 (58.1)
Patient indicates that they do not use their medication at times	1 (33.3)	14 (31.1)
>0 prescriptions of systemic corticosteroids	1 (33.3)	12 (26.7)
History of intubation	0 (0)	5 (11.1)
Inhalation technique suboptimal	0 (0)	4 (8.8)
>0 unscheduled attendance, admission, or visit	0 (0)	3 (6.7)

**Fig. 2:**
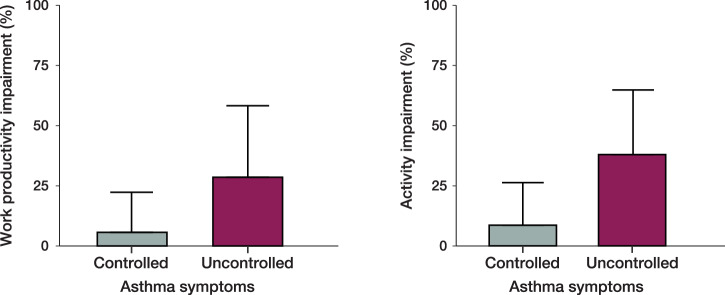
Work productivity and activity impairment in patients with controlled versus uncontrolled asthma. Work productivity impairment (%) indicates how much work was missed because of health problems and how much work time was impaired while working. Activity impairment (%) indicates impairment in daily activities, irrespective of work, due to health problems. Bar plots show mean and standard deviation.

Patients with uncontrolled asthma experienced higher levels and greater odds of work productivity impairment as measured with the WPAI tool (OR, 1.05 per % increase in reduced productivity; 95% CI, 1.03–1.09; *p* < 0.01) and higher levels and greater odds of impairments in their daily activities (OR, 1.07 per % increase in impairment; 95% CI, 1.05–1.09; *p* < 0.01; Fig. 2).

Mean (SD) bEOS counts for patients with controlled versus uncontrolled asthma were 258 (182) cells/µL and 220 (159) cells/µL, respectively. Patients with uncontrolled asthma were not at greater odds of having a higher bEOS count than patients with controlled asthma (OR, 0.39; 95% CI, 0.06–2.59; *p* = 0.32).

### Usability results

A total of 33 GPNs and 1 general practitioner participated in the qualitative survey concerning feedback on the AsthmaOptimiser tool. The GPNs were all women, and the general practitioner was male. The respondents’ mean (SD) age was 46.5 (9.4) years, and 11 respondents had missing data. The mean (SD) length of working experience was 11 (8.9) years, with 16 respondents having missing data. The mean (SD) number of hours worked per week for the providers was 25.9 (6.23) hours, with 12 respondents having missing data.

General feedback obtained from the face-to-face interviews with the healthcare providers was grouped as follows: (1) perceptions of the tool before and after use, including positive and negative feedback; (2) AsthmaOptimiser content; and (3) areas of improvement. Prior to using the tool, respondents provided insights about the potential benefits of the AsthmaOptimiser. They felt the tool could be a good addition to asthma consultations, a way to obtain advice for treatment, a way to get advice based on input, and a way to gain insights into whether the patients were doing as well as the providers thought they were. A comment not directly related to the tool addressed current issues that providers face, specifically that it was hard to create an overview of patients with asthma, help patients keep proper control of their asthma, and not have influence on whether their patients come in for annual reviews.

Feedback from providers indicated that they found it easy to log in to the AsthmaOptimiser, the guidance was clear, and patients could complete the ACQ-6^23^ themselves. The most frequent negative feedback was that the tool was time-consuming and could not store all patient data (Table 3). Some of the practitioners had difficulty finding information and felt it was difficult to navigate between areas within the AsthmaOptimiser tool.

**Table 3:** AsthmaOptimiser usability: feedback from 14 responders.

	Respondent feedback	Number of respondents (N = 14)
**Positive comments**	Logging in was easy; guidance was clear; consultation was step-by-step or aligned with normal consultation process.	4
	Patients could fill out the ACQ-6 themselves; patients were willing to participate; it was easy to include patients.	3
	Respondents were pleased with different sections or questions of the tool, e.g., inhaler section, risk assessment questions, summary, comorbidities, and socioeconomic questions.	2
	It was easy to skip a section.	1
	The equipment worked as expected, e.g., laptop and phone worked without any problems.	1
	The electronic medical record number only needed to be entered once because the system remembered it.	1
	Optimiser has added value compared with the HIS protocol, which does not give advice.	1
**Negative comments**	The tool was time-consuming or did not capture all patient data, e.g., test values and medications; “spent half hour on consultation with the tool”; “continuously going back to the screen”; “EMR link has to be good.”	6
	Free text cannot be added to “other” choices or answer options are lacking, e.g., “does not apply.”	5
	The tool does not capture all data, e.g., medications; it is difficult to find some items in the tool.	3
	It is difficult to move back and forth in the tool.	3
	The aim of the tool should be made clear, e.g., “what do you want to achieve by implementing this tool?”	1
	It is difficult to find information online about AsthmaOptimiser (the website has a different name).	1
	The ordering of sections in the tool does not appeal.	1
	The summary is too long.	1

*ACQ-6* Asthma Control Questionnaire 6 items, *EMR* electronic medical record, *HIS* health information system.

Half of the respondents who provided feedback on the AsthmaOptimiser content identified the inability to add free text to fields marked “other” and requested the inclusion of more follow-up questions (e.g., type of work, how many hours worked, or asthma triggers; Supplemental Table S2). It is worthwhile to note that such free text boxes were not included due to the possibility of writing identifiable patient information in these boxes, which would put additional General Data Protection Regulation (GDPR) requirements on the tool. Additionally, because the AsthmaOptimiser was hosted on AstraZeneca servers and required them to report any adverse events, the option for adding free text was not included in the development. Following user feedback and the transfer of the tool outside AstraZeneca, this has been added to the tool. The respondents also raised questions about the order of some of the questions. For example, questions on exacerbations and lung function tests came before the inhaler technique section.

The respondents also inquired about expanding the amount of information gathered on medication use questions to get a clearer perspective on patient adherence and entering inhaler prescriptions and the number of inhalers patients used. The respondents also noted inconsistencies in how single inhaler prescriptions and the number of inhalers used per year were recorded. Each prescription was interpreted by the tool as 1 inhaler, whereas many patients often pick up multiple inhalers on a single prescription. Another limitation involved a video on inhaler technique. Sometimes, the video worked well; however, in some cases, it stopped working. Overall, the nurses felt that the tool and the advice it provided were useful.

Providers also reported their appreciation of the advice to lower the dose of inhaled corticosteroids. This advice helped 1 provider think about this issue and made it easier to convince the patient to lower their medication dosage. Another provider shared that the suggestions from the AsthmaOptimiser about increasing the dosage of inhaled corticosteroids were seen as a confirmation of their own decision-making abilities. Additional comments about the content focused on receiving more information about ACQ-6 scores and interpretation of or advice about the results, identifying challenges with accounting for medications that started halfway through the year and the difficulty of transitioning to the risk assessment.

Provider feedback on areas of improvement overlapped with comments made about the usability and content of the tool (Supplemental Table S3). A few providers indicated they were waiting for additional features, e.g., self-management plan and traffic light functionality to identify important items to discuss with patients. Discrepancies between data stored in electronic medical records and answers to questions (e.g., smoking may have been a trigger, but the patient’s medical record indicates the patient smokes, which was not true) was another concerning issue raised, along with suggestions to revise the risk factor assessment questions.

## Discussion

The use of the AsthmaOptimiser in the CAPTURE study showed that 60% of patients with asthma attending primary care annual asthma reviews had uncontrolled asthma. Opportunities to improve asthma control during the consultations offered guidance on best practices (e.g., education on inhaler technique or medication adherence) to general practitioners. Women made up a larger percentage of the patients with uncontrolled asthma and patients with uncontrolled asthma were at greater odds of experiencing impairments in work productivity and daily activities.

Patients with uncontrolled asthma made up most referral recommendations in this study, yet most of these patients did not have a referral scheduled. The information provided by the AsthmaOptimiser based on the GINA guidelines may have caused providers to consider other solutions to address uncontrolled asthma. Conversations between GPNs and patients, coupled with a review of their medical information, provided reasons for not following the advice for a referral to a specialist. Providers who identified patients who needed education and training to improve inhalation technique, medication adherence, or both, discussed other treatment options (e.g., referral to a physiotherapist for breathing exercises) and addressed lifestyle changes (e.g., smoking cessation).

Improvements in medication adherence and inhaler technique through patient education have been shown to improve asthma management^24–26^, reduce asthma exacerbations, and result in the use of fewer oral corticosteroids^1,27–29^. Behavioural therapy aimed at medication adherence, proper inhalation technique, and lifestyle changes educates patients and engages them in shared decision-making, especially for patients who do not want a referral to a specialist^24^. While the asthma control of many patients can be optimised in general practice for specific patients (e.g., persistent uncontrolled asthma with ≥2 exacerbations per year), referral to a specialist is a viable treatment option and therefore, should not be ignored; for example, additional follow-ups are important in patients who do not show improvements in symptoms after initial adjustments following a consultation with the AsthmaOptimiser.

Healthcare providers who used this tool in the CAPTURE study were asked to evaluate the tool’s usability and content, as well as to identify areas of improvement. Providers found the tool easy to use because it aligned with the normal consultation process, worked as expected, and was linked to other electronic systems. Learning a new tool and software program does present some challenges. Working through the tool required time that some providers felt was diverting their attention from their patients. The AsthmaOptimiser also had limitations in navigation within the tool and in not capturing all patient data that each provider would have preferred (e.g., a “free text” box where additional information could be stored). However, the capacity to store data was guided by adherence to the GDPR and limited due to regulation by the study funder^30^. The time constraints identified by some of the nurses may have been related to learning a new software program. Using new, unfamiliar software usually takes more time initially^31^. It is anticipated that as the learner becomes more acclimated with the content and functionality of the tool, the amount of time needed to enter information will be reduced^31^.

A strength of this study is the implementation of the AsthmaOptimiser tool, which allowed us to determine adherence to asthma management guidelines and identify areas where changes in the tool could add to patient information to align care better. A limitation of this study includes a potential selection bias arising from a convenience sample (i.e., patients who had regular asthma consultations were invited to participate in the study).

Following the feedback from this study, ownership of the AsthmaOptimiser tool was transferred out of the sponsor and the feedback from the users was used to create a newer version following the most recent update to GINA recommendations. The AsthmaOptimiser is registered as a Class IIa medical device (UDI-ID: 4270001023551) and is available from www.asthmaoptimiser.com.

In conclusion, the majority of patients attending annual asthma visits in Dutch primary care had uncontrolled asthma. Women and individuals with lower socioeconomic status were identified as being at greater odds of having uncontrolled asthma; however, the odds of having uncontrolled asthma decreased with age. Patients with uncontrolled asthma experienced higher levels and greater odds of impairment in work productivity and daily activities. Information from the AsthmaOptimiser revealed opportunities to optimise asthma management in primary care and identified patients in need of a referral based on GINA recommendations. Even though new software (i.e., the AsthmaOptimiser tool) can be time-consuming to learn and use, overall, the GPNs found the AsthmaOptimiser to be easy to use and a good addition to asthma consultations and provided suggestions for future enhancements.

## Supplementary information

Supplementary Information

## Data Availability

The datasets used and/or analysed during the current study available from the corresponding author on reasonable request.

## References

[1] Global Initiative for Asthma. *Global strategy for asthma management and prevention*, https://ginasthma.org/wp-content/uploads/2022/05/GINA-Main-Report-2022-FINAL-22-05-03-WMS.pdf (2022).

[2] GBD 2016. Disease and Injury Incidence and Prevalence Collaborators. Global, regional, and national incidence, prevalence, and years lived with disability for 328 diseases and injuries for 195 countries, 1990-2016: a systematic analysis for the Global Burden of Disease Study 2016. *Lancet* **390**, 1211–1259 (2017). 10.1016/S0140-6736(17)32154-2 PMC5605509 28919117

[3] Chung, KF et al. International ERS/ATS guidelines on definition, evaluation and treatment of severe asthma. *Eur. Respir. J.* **43**, 343–373 (2014). 24337046 10.1183/09031936.00202013

[4] Cataldo, D et al. Severe asthma: oral corticosteroid alternatives and the need for optimal referral pathways. *J. Asthma* **58**, 448–458 (2020). 31928102 10.1080/02770903.2019.1705335

[5] Foster, JM, McDonald, VM, Guo, M & Reddel, HK “I have lost in every facet of my life”: the hidden burden of severe asthma. *Eur. Respiratory J.* **50**, 1700765 (2017). 10.1183/13993003.00765-2017 28931662

[6] Menzies-Gow, A & Chiu, G Perceptions of asthma control in the United Kingdom: a cross-sectional study comparing patient and healthcare professionals’ perceptions of asthma control with validated ACT scores. *NPJ Prim. Care Respir. Med* **27**, 48 (2017). 28801654 10.1038/s41533-017-0050-x PMC5554258

[7] GBD 2015. Chronic Respiratory Disease Collaborators. Global, regional, and national deaths, prevalence, disability-adjusted life years, and years lived with disability for chronic obstructive pulmonary disease and asthma, 1990-2015: a systematic analysis for the Global Burden of Disease Study 2015. *Lancet Respir. Med* **5**, 691–706 (2017). 10.1016/S2213-2600(17)30293-X PMC5573769 28822787

[8] Global Asthma Network. *The global asthma report*, http://globalasthmareport.org/2014/Global_Asthma_Report_2014.pdf (2014).

[9] Caminati, M, Vaia, R, Furci, F, Guarnieri, G & Senna, G Uncontrolled asthma: unmet needs in the management of patients. *J. Asthma Allergy* **14**, 457–466 (2021). 33976555 10.2147/JAA.S260604 PMC8104981

[10] Price, D, Fletcher, M & van der Molen, T Asthma control and management in 8,000 European patients: the REcognise Asthma and LInk to Symptoms and Experience (REALISE) survey. *NPJ Prim. Care Respir. Med* **24**, 14009 (2014). 24921985 10.1038/npjpcrm.2014.9 PMC4373302

[11] von Bulow, A et al. Differentiation of adult severe asthma from difficult-to-treat asthma - outcomes of a systematic assessment protocol. *Respir. Med* **145**, 41–47 (2018). 30509715 10.1016/j.rmed.2018.10.020

[12] Price, D, Bjermer, L, Bergin, DA & Martinez, R Asthma referrals: a key component of asthma management that needs to be addressed. *J. Asthma Allergy* **10**, 209–223 (2017). 28794645 10.2147/JAA.S134300 PMC5536139

[13] Royal College of Physicians. *Why asthma still kills. The National Review of Asthma Deaths (NRAD)*, (2014).

[14] Beekman, M, Hales, J, Al-Ahmad, M, del Olmo, R & Tze, TL Breaking the vicious circle – the Asthma Referral Identifier (ReferID) tool. *NPJ Prim. Care Respir. Med* **32**, 40 (2022). 36209272 10.1038/s41533-022-00296-6 PMC9547879

[15] *ClinicalTrials.gov identifier NCT04941001. Bethesda MD: National Institutes of Health*, https://clinicaltrials.gov/ct2/show/NCT04941001 (2022).

[16] *Optimisation of ASthma in Those with Uncontrolled Symptoms (OASIS)*, https://ichgcp.net/clinical-trials-registry/NCT04941001 (2022).

[17] Dhruve, H, Nanzer, A & Jackson, DJ Efficacy of the referID+ digital tool in uncontrolled asthma in primary care (OASIS): a randomised controlled trial. *Thorax* **78**, S90 (2023).

[18] *ClinicalTrials.gov identifier NCT04456270. Bethesda, MD: National Institutes of Health*, https://clinicaltrials.gov/ct2/show/NCT04456270 (2020).

[19] Reilly, MC, Zbrozek, AS & Dukes, EM The validity and reproducibility of a work productivity and activity impairment instrument. *Pharmacoeconomics* **4**, 353–365 (1993). 10146874 10.2165/00019053-199304050-00006

[20] Bates, D., Machler, M., Bolker, B. M. & Walker, S. C. Fitting linear mixed-effects. *J. Stat Softw* **67**, 1–48 (2015).

[21] R: a language and environment for statistical computing. (R Foundation for Statistical Computing, Vienna, Austria, 2008).

[22] Rstudio: integrated development for R. (PBC, Boston, MA, 2020).

[23] American Thoracic Society. *Asthma Control Questionnaire (ACQ)*.

[24] George, M, Graff, C, Bombezin-Domino, A & Pain, E Patients with severe uncontrolled asthma: perception of asthma control and its management. *Pulm. Ther.* **8**, 209–223 (2022). 35471688 10.1007/s41030-022-00190-z PMC9098739

[25] National Asthma Education and Prevention Program Coordinating Committee. *Expert Panel Report 3 (EPR-3): Guidelines for the diagnosis and management of asthma-summary report*, (2007). 10.1016/j.jaci.2007.09.043 17983880

[26] Hannane, A, Misane, L, Devouassoux, G, Colin, C & Letrilliart, L Asthma patients’ perception on their care pathway: a qualitative study. *NPJ Prim. Care Respir. Med* **29**, 9 (2019). 30940806 10.1038/s41533-019-0121-2 PMC6445145

[27] Makhinova, T, Barner, JC, Richards, KM & Rascati, KL Asthma controller medication adherence, risk of exacerbation, and use of rescue agents among Texas Medicaid patients with persistent asthma. *J. Managed Care Specialty Pharm.* **21**, 1124–1132 (2015). 10.18553/jmcp.2015.21.12.1124 PMC10401995 26679962

[28] Pollard, S, Bansback, N, FitzGerld, JM & Bryan, S The burden of nonadherence among adults with asthma: a role for shared decision-making. *Allergy* **72**, 705–712 (2017). 27873330 10.1111/all.13090

[29] AL-Jahdali, H et al. Improper inhaler technique is associated with poor asthma control and frequent emergency department visits. *Allergy, Asthma Clin. Immunol.* **9**, 1–7 (2013). 23510684 10.1186/1710-1492-9-8 PMC3605255

[30] Proton Technologies AG. *Complete guide to GDPR compliance*, (2023).

[31] Zaman, N, Goldberg, DM, Kelly, S, Russell, RS & Drye, SL The relationship between nurses’ training and perceptions of electronic documentation systems. *Nurs. Rep.* **11**, 12–27 (2021). 34968308 10.3390/nursrep11010002 PMC8608127

