# Supplementary material for: Asthma control and opportunities to optimize management and the healthcare provider experience using the AsthmaOptimiser online tool in Dutch general practice: the CAPTURE study
Source: NPJ Prim Care Respir Med. 2025 Apr 15;35:23. doi: 10.1038/s41533-025-00427-9 (PMC11997121; doi:10.1038/s41533-025-00427-9)
Supplement: Supplementary file 1 — Supplementary Information [file 41533_2025_427_MOESM1_ESM.pdf]

**Asthma control and opportunities to optimize management and the healthcare provider experience using the AsthmaOptimiser online tool in Dutch general practice: The CAPTURE Study**

Marika T. Leving[1], Yoran H. Gerritsma[1], David J. Jackson[2,3], Erik W.M.A. Bischoff[4], Jiska Meijer[1], Hans Wouters[1], Bertine Flokstra-de Blok[1,5,6], & Janwillem Kocks[1,5,7,8*]

[1]General Practitioners Research Institute, Groningen, The Netherlands

[2]Guy's Severe Asthma Centre, Guy's & St Thomas' NHS Trust, London, United Kingdom

[3]School of Immunology & Microbial Sciences, King's College London, London, United Kingdom

[4]Department of Primary and Community Care, Radboud Institute for Health Sciences, Radboud University Medical Centre, Nijmegen, The Netherlands

[5]University of Groningen, University Medical Center Groningen, GRIAC Research Institute, Groningen, The Netherlands

[6]University of Groningen, University Medical Center Groningen, Beatrix Children's Hospital, Department of Pediatric Pulmonology and Pediatric Allergology, Groningen, The Netherlands

[7]Observational and Pragmatic Research Institute, Singapore

[8]Department of Pulmonology, University of Groningen, University Medical Center Groningen, Groningen, The Netherlands

**Supplement S1**.

The GPNs offered explanations for why 32 of 41 patients with available information who were identified for a referral to a specialist did not receive a referral in the 6 months after the visit (**Supplemental Table S1**). The explanations were grouped by considerations not to follow the advice for referring the patient to a specialist (e.g., improvements in medication adherence and inhaler technique) and reasons for not scheduling referrals to a specialist (e.g., reduction of symptoms, changes in medications, or patient referral to a cardiologist because of shortness of breath).

**Table S1.** General practitioner perspective on specialist referral and referral outcomes[a]

| **General practitioner actions** | **Explanations associated with actions** |
|---|---|
| Consideration to not follow advice to schedule referral to specialist | Inhalation technique or medication adherence improved<br>Other treatment options pursued, e.g., breathing exercises or lifestyle or smoking advice<br>Patient did not want to be referred |
| Actions providers took | Discussed medication adherence<br>Trained patient in inhalation technique<br>Discussed lifestyle, sanitation, smoking cessation<br>Referred for consultation with lung function test |
| **Patient referral status** | **Outcome** |
| Referred to a specialist | Patient referred to a specialist (n=8)<br>Unknown (n=1) |
| Reasons no referrals were scheduled | Symptoms reduced (n=12)<br>Other reason not specified (n=6) |

| | |
|---|---|
| | Patient did not want to be referred (n=4) |
| | Patient not (yet) seen again (n=4) |
| | Other medication or increase in medication (n=2) |
| | Patient moved (n=1) |
| | 2 courses of antibiotics had been prescribed (n=1) |
| | General practitioner treated lung attacks (n=1) |
| | Referred to cardiologist because of shortness of breath (n=1) |

[a]Information was available for follow-up for 41of 48 patients.

**Table S2.** AsthmaOptimiser content: feedback from 10 responders

| Respondent feedback | Number of respondents (N=10) |
|---|---|
| Free text cannot be added to "other" choices or answer options, e.g., the "does not apply" option is lacking. | 5 |
| Follow-up questions or additional fields are needed, e.g., to ask follow-up questions about work, such as whether complaints are worse, type of job, and how many hours worked; to ask about asthma triggers; "medication answer 'not always' does not get all possible reasons." | |
| The prescription field should allow the user to enter the number of devices or number of prescriptions per year; the current format is not useful for patients with multiple devices on a single prescription. | 3 |
| The tool has electronic challenges, e.g., the end of the inhaler technique section video was "a bit treacherous"; "the video works fine, but after a while, it stopped working." | 3 |
| The summary is clear and can justify scheduling a referral. | 3 |
| The order of questions is not optimal, e.g., it starts with a question on exacerbations; lung function test results come before the inhaler technique section. | 2 |
| Skip logic is missing in some data fields. | |
| The inhaler technique section is good. | 2 |
| "Summary was lengthy, prefer to have it accessible sooner"; "there is added value if everything is properly linked." | 2 |
| The referral letter should be copied to the company that connects healthcare providers to patients through digital patient referrals; "currently, it is a nuisance." | |
| There are discrepancies between the recommendations and the provider's or patient's perceptions, e.g., referrals. | 2 |

| | |
|---|---|
| "Advice to lower ICS is great"; "helps provider start thinking about it and becomes easier to convince the patient to lower their ICS"; "if Optimiser advises it, it must be true."<br>Advice to increase ICS is good, e.g., "I would have done that myself as well"; "was a confirmation of my own actions, which was pleasant." | 2 |
| Providers sometimes follow what the tool advises or receive confirmation of their own actions. | 2 |
| Patients found it very useful to get into more detail and appreciated it. | 2 |
| More information is needed, e.g., ACQ-6 scores, text, and what scores mean. | 1 |
| Advice to improve adherence was received often, even if the provider deemed the patient to be adherent, "which was a shame." | 1 |
| Asking for $FEV_1$ and FVC in litres is "a bit limited"; No option is given for pre- and post measurements. | 1 |
| Sections are easy to skip. | 1 |
| The transition to risk factor assessment is difficult. | 1 |
| The risk factor assessment was liked. | 1 |
| There are issues with discrepancies between the information entered in the tool [and timing], e.g., medication started halfway through the year.<br>The tool was wrong, i.e., "change in use overtime was not an option." | 1 |

*ACQ-6*, Asthma Control Questionnaire, 6 items; *$FEV_1$*, first second of forced expiration; *FVC*, forced vital capacity; *ICS*, inhaled corticosteroid.

**Table S3.** Areas of improvement with the AsthmaOptimiser: feedback from 7 responders

| Respondent feedback | Number of respondents, (N=7) |
|---|---|
| Questions are missing, e.g., length and weight, ACQ-6 scores for each per question, and smoking behaviour; "would like to see ACQ-6 text in tool with explanation of results and advice based on ACQ-6." | 2 |
| Additional features that can be collapsed are needed, e.g., a self-management plan and traffic light ("important to discuss with patients") and a checklist with washing hands. | 2 |
| Moving back and forth in the tool is difficult. | 1 |
| There are discrepancies between the data collected and answers to questions, e.g., "smoking may have been a trigger, but the patient's medical record indicates the patient smokes, which was not true." | 1 |
| Similar results are in more than 1 location, e.g., "useful to have all values from the lung function test in 1 place, had to enter it twice in our own system and in the tool because the tool could only fill in the $FEV_1$ and FVC." | 1 |
| Risk factor assessment questions could be revised, e.g., "ask whether environment/compounds make people experience more difficulty with asthma"; "air pollution was too broad"; "vaping was an unknown word, maybe add electronic smoking"; "cleaning products were not an option"; "socioeconomic status was difficult"; "living conditions were vague." | 1 |
| Information is needed on what the tool includes, e.g., "would be useful to understand what values were calculated in the tool and whether anything will be added later." | 1 |
| The tool should consider questions for patients with health literacy issues. | 1 |

*ACQ-6*, asthma control questionnaire 6 items; *$FEV_1$*, forced expired volume in 1 second; *FVC*, forced vital capacity.

GP
RI

# Supplemental Appendix: AsthmaOptimiser example case from CAPTURE study

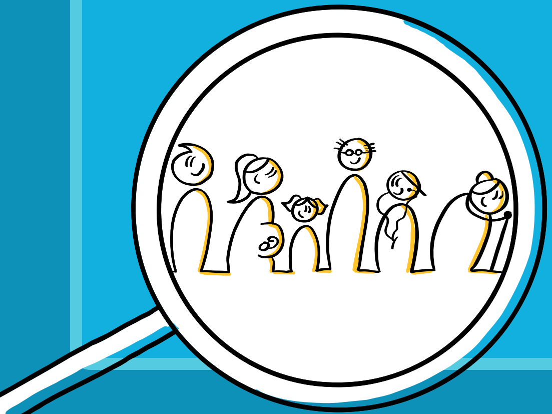

Making research participation an enjoyable experience

2

# Case

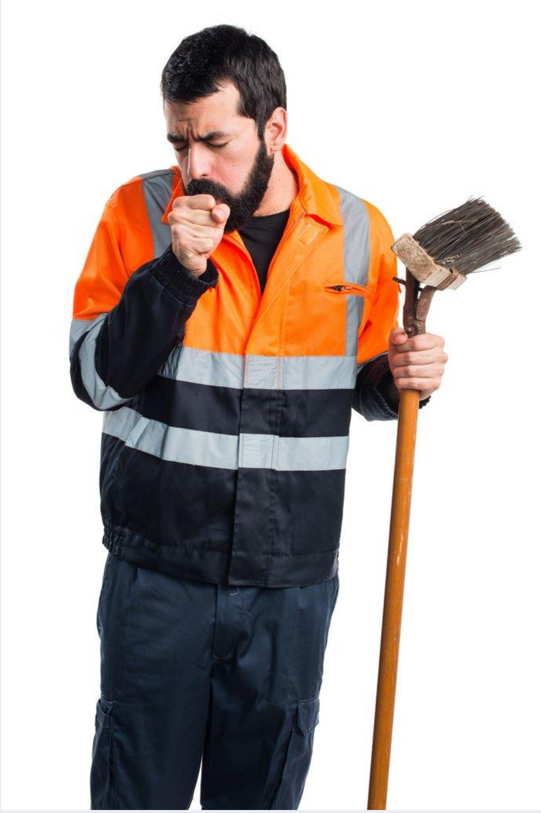

- Male
- 39 years old
- 173 cm
- 71 kg
- Annual review consultation at the nurse

3

Asthma Optimiser

SECTIONS

- Exacerbation history
- Symptom control
- Adherence & attitudes
- Inhaler technique
- Risk factor assessment
- Lung function test

OUTPUT

- Summary

Support

Terms & Conditions

Privacy Policy

Disclaimer

< Back

SYSTEMIC CORTICOSTEROIDS

## How many prescriptions of systemic corticosteroids has the patient received for asthma over the past 12 months?

3 prescriptions

Confirm

Additional info

At least 1 severe exacerbation in the last 12 months is a major independent risk factor for future exacerbations. The need for long-term oral corticosteroid (OCS) use and/or frequent courses of OCS (e.g. two or more courses per year) is an indication that the patient may benefit from a review by a specialist.

Please refer to the GINA 2023 report for additional information.

Increasing cumulative exposure and increasing mean daily exposure to SCS places patients at a high risk of potentially debilitating adverse outcomes. The onset of some outcomes was associated with exposure to only four lifetime courses of SCS.

A Charter to Improve Patient Case in Severe Asthma

Adverse Outcomes from Initiation of Systemic Corticosteroids for Asthma: Long-term Observational Study

Additional information available in drop down menu

GP RI

4

Asthma Optimiser

‹ Back

SECTIONS

- Exacerbation history
- Symptom control
- Adherence & attitudes
- Inhaler technique
- Risk factor assessment
- Lung function test

OUTPUT

- Summary

EMERGENCY VISITS

# How many times has the patient had an emergency attendance, admission or unscheduled visit due to asthma over the past 12 months?

- (●) Never
- ( ) 1 time
- ( ) 2 times or more

Additional info

Frequent asthma-related health care utilisation (e.g. multiple emergency department visits or urgent primary care visits) is an indication that the patient may benefit from a review by a specialist.

Please refer to the GINA 2023 report for additional information.

Support

Terms & Conditions

Privacy Policy

Disclaimer

GP RI

5

Asthma Optimiser

SECTIONS

Exacerbation history

Symptom control

Adherence & attitudes

Inhaler technique

Risk factor assessment

Lung function test

OUTPUT

Summary

Support

Terms & Conditions

Privacy Policy

Disclaimer

< Back

INTENSIVE CARE

## Has the patient ever been intubated or admitted to an ICU (intensive care unit) or a high dependency unit due to their asthma?

Yes No

Additional info

Being intubated or admitted to an ICU are major independent risk factors for future exacerbations. Near-fatal asthma attack (ICU admission, or mechanical ventilation for asthma) at any time in the past is an indication that the patient may benefit from a review by a specialist.

Please refer to the GINA 2023 report for additional information.

ASTHMA AT WORK

GP RI

6

Asthma Optimiser

SECTIONS

- Exacerbation history
- Symptom control
- Adherence & attitudes
- Inhaler technique
- Risk factor assessment
- Lung function test

OUTPUT

- Summary

Support

Terms & Conditions

Privacy Policy

Disclaimer

< Back

ASTHMA AT WORK

# Are the patient's asthma symptoms worse when the patient is at work? (i.e. symptoms are better at home)

Select no if the patient is not working, is retired or works from home.

Yes No

Additional info

Suspected occupational asthma is an indication that the patient may benefit from a review by a specialist for confirmatory testing and identification of the sensitising or irritant agent, specific advice about eliminating exposure and pharmacological treatment.

Please refer to the GINA 2023 report for additional information.

GPRI

7

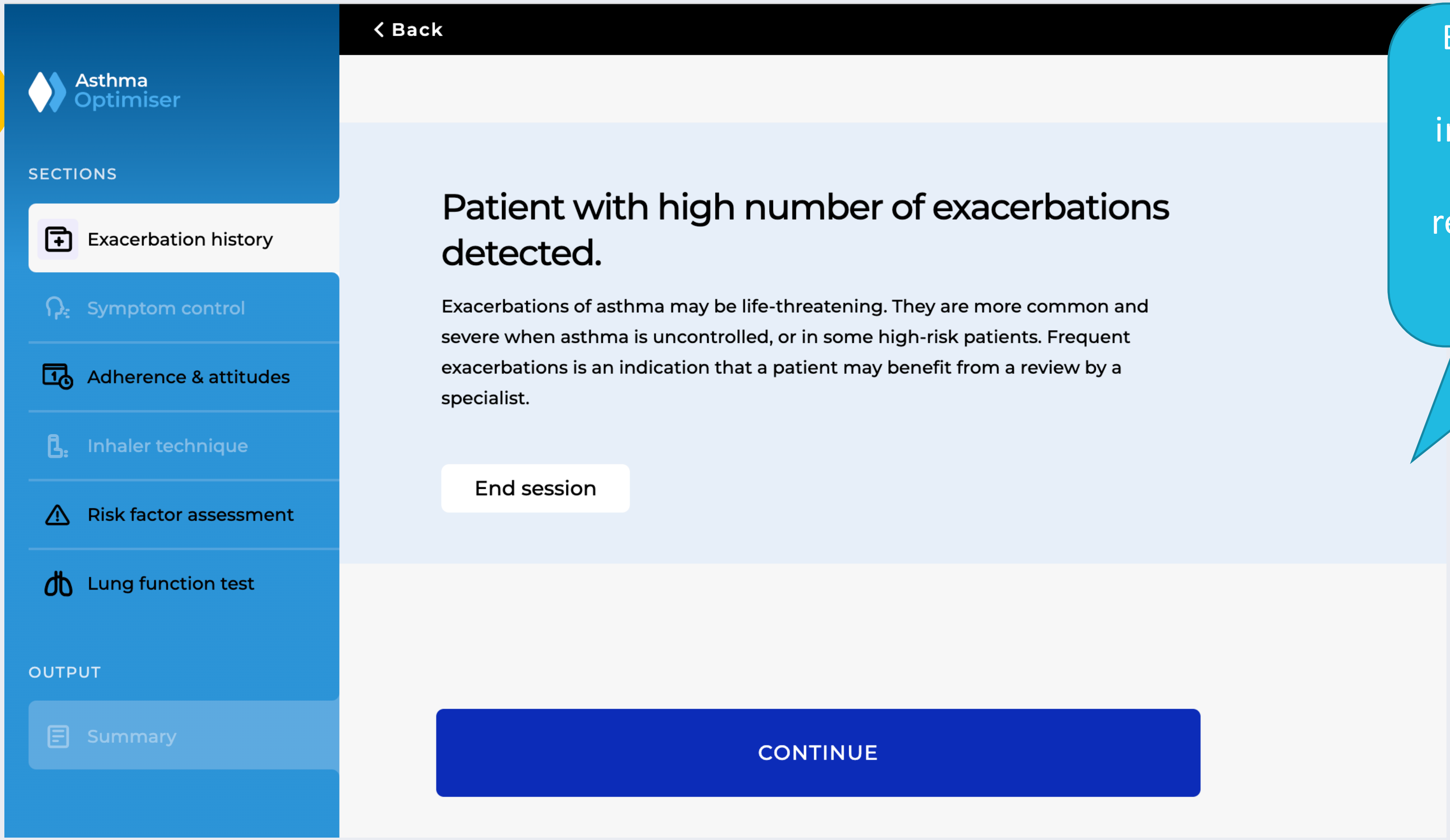


Based on this small amount of information. Patient should already be referred to specialist according to the guidelines.

8

Asthma Optimiser

SECTIONS

- Exacerbation history
- Symptom control
- Adherence & attitudes
- Inhaler technique
- Risk factor assessment
- Lung function test

OUTPUT

- Summary

Support

Terms & Conditions

Privacy Policy

Disclaimer

< Back

ACQ6 QUESTIONNAIRE

# We will now record the patient's symptom control using ACQ6 as a guideline.

If you use the ACQ6 questions please make sure you have them to hand and go over them with your patient.

If you don't have a printed version, click here for a pdf version. On the next page, you'll see numbers that correspond with the questionnaire. Simply mark the number of their response that best describes how they've been feeling **in the past week**.

CONTINUE

GPRI

Back

Asthma Optimiser

SECTIONS

- Exacerbation history
- Symptom control
- Adherence & attitudes
- Inhaler technique
- Risk factor assessment
- Lung function test

OUTPUT

- Summary

ACQ6 QUESTIONNAIRE

# Please select the relevant answers for the ACQ6 questions below:

Question 1 - On average, during the past week, how often was the patient woken by his/her asthma during the night?

0 1 2 3 4 5 6

Question 2 - On average, during the past week, how bad were the patient's asthma symptoms when he/she woke up in the morning?

0 1 2 3 4 5 6

Question 3 - In general, during the past week, how limited was the patient in his/her activities because of his/her asthma?

0 1 2 3 4 5 6

Question 4 - In general, during the past week, how much shortness of breath did the patient experience because of his/her asthma?

0 1 2 3 4 5 6

Question 5 - In general, during the past week, how much of the time did the patient wheeze?

0 1 2 3 4 5 6

Question 6 - On average, during the past week, how many puffs of short-acting bronchodilator has the patient used each day?

0 1 2 3 4 5 6

ACQ Score: 1.0 (Uncontrolled asthma)

Additional info

CONTINUE

RI

10

Back

Asthma Optimiser

SECTIONS

- Exacerbation history
- Symptom control
- Adherence & attitudes
- Inhaler technique
- Risk factor assessment
- Lung function test

OUTPUT

- Summary

Support

Terms & Conditions

Privacy Policy

Disclaimer

INHALER TYPE

# Which inhaler type is the patient prescribed?

- ( ) Reliever
- ( ) Controller
- (•) Both

Additional info

Reliever (rescue) medications are provided to all patients for as-needed relief of breakthrough symptoms, including during worsening asthma or exacerbations. They are also recommended for short-term prevention of exercise-induced bronchoconstriction (EIB). Relievers include the anti-inflammatory relievers ICS-formoterol and ICS-SABA, and SABA alone.

In the past, the term controller medications mostly referred to medications containing ICS that were used to reduce airway inflammation, control symptoms, and reduce risks such as exacerbations and related decline in lung function. In GINA Track 1, controller treatment is delivered through an anti-inflammatory reliever (AIR), low-dose ICS-formoterol, taken when symptoms occur and before exercise or allergen exposure; in Steps 3-5, the patient also takes maintenance controller treatment (daily or twice-daily ICS-formoterol). This is called maintenance-and-reliever therapy (MART).

Select "both" if the patient is prescribed Maintenance and Reliever Therapy.

Please refer to the GINA 2023 report for additional information.

GP RI

11

Asthma Optimiser

SECTIONS

- Exacerbation history
- Symptom control
- Adherence & attitudes
- Inhaler technique
- Risk factor assessment
- Lung function test

OUTPUT

- Summary

Support

Terms & Conditions

Privacy Policy

Disclaimer

< Back

ADDITIONAL MEDICATION

## Does the patient use one of the following medications?

- [ ] Long-acting muscarinic antagonist (LAMA)
- [ ] Maintenance oral corticosteroids (OCS)
- [ ] Biological treatment
- [ ] Leukotriene receptor antagonist (LTRA)
- [x] None

Additional info

Additional treatment with LAMA, maintenance OCS, or biological treatment is assigned to GINA treatment step 5. Please refer to page 65 of the GINA 2023 report for additional information.

Important: The Asthma Optimiser does not support further treatment assessment if the patient is in GINA treatment step 5.

CONTINUE

GP RI

12

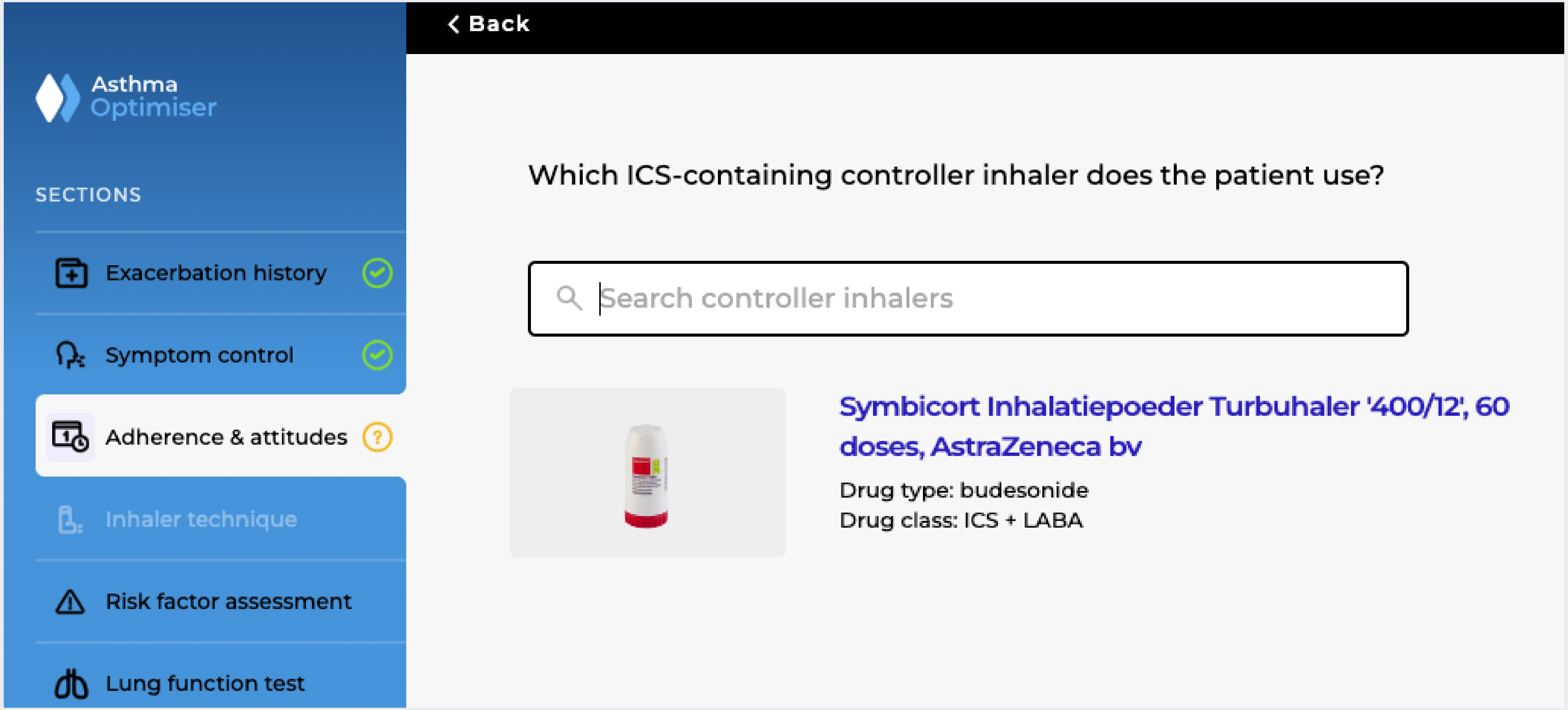

Back
Asthma Optimiser
SECTIONS
Exacerbation history
Symptom control
Adherence & attitudes
Inhaler technique
Risk factor assessment
Lung function test
Which ICS-containing controller inhaler does the patient use?
Search controller inhalers
Symbicort Inhalatiepoeder Turbuhaler '400/12', 60 doses, AstraZeneca bv
Drug type: budesonide
Drug class: ICS + LABA


GP RI

13

Asthma Optimiser

SECTIONS

- Exacerbation history
- Symptom control
- Adherence & attitudes
- Inhaler technique
- Risk factor assessment
- Lung function test

OUTPUT

- Summary

‹ Back

CONTROLLER DOSAGE

## What is the controller dosage and inhalations taken by the patient per day?

320 micrograms per inhalation, 2 inhalation(s) per day.

Confirm

365*2dd = 730 inhalations per year

Additional info

The prescribed dosage of the controller medication, together with the number of controller prescriptions over the past 12 months, can be used to estimate treatment adherence.

GPRI

14

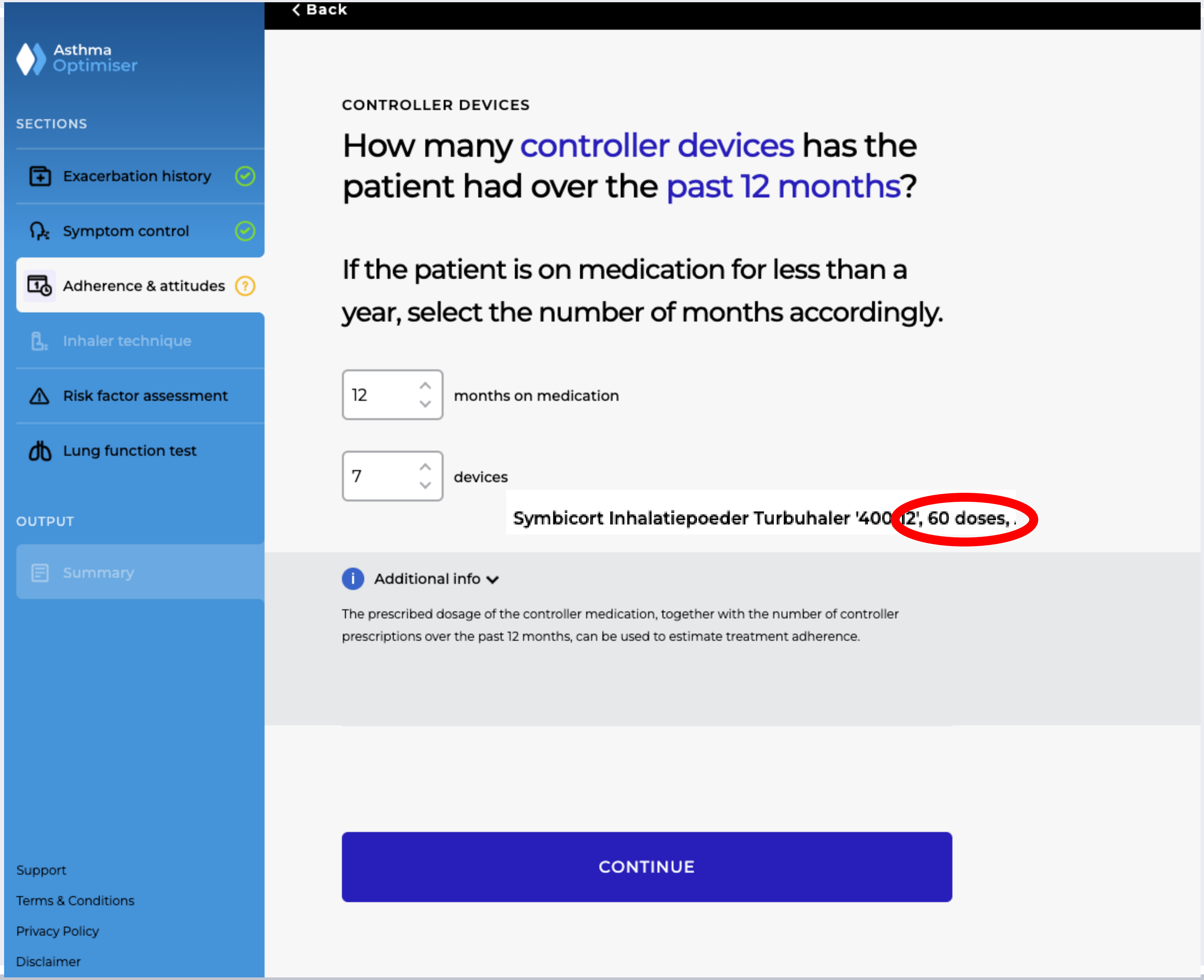


60*7 = 420 doses picked up

420/730 = 57% adherence

15

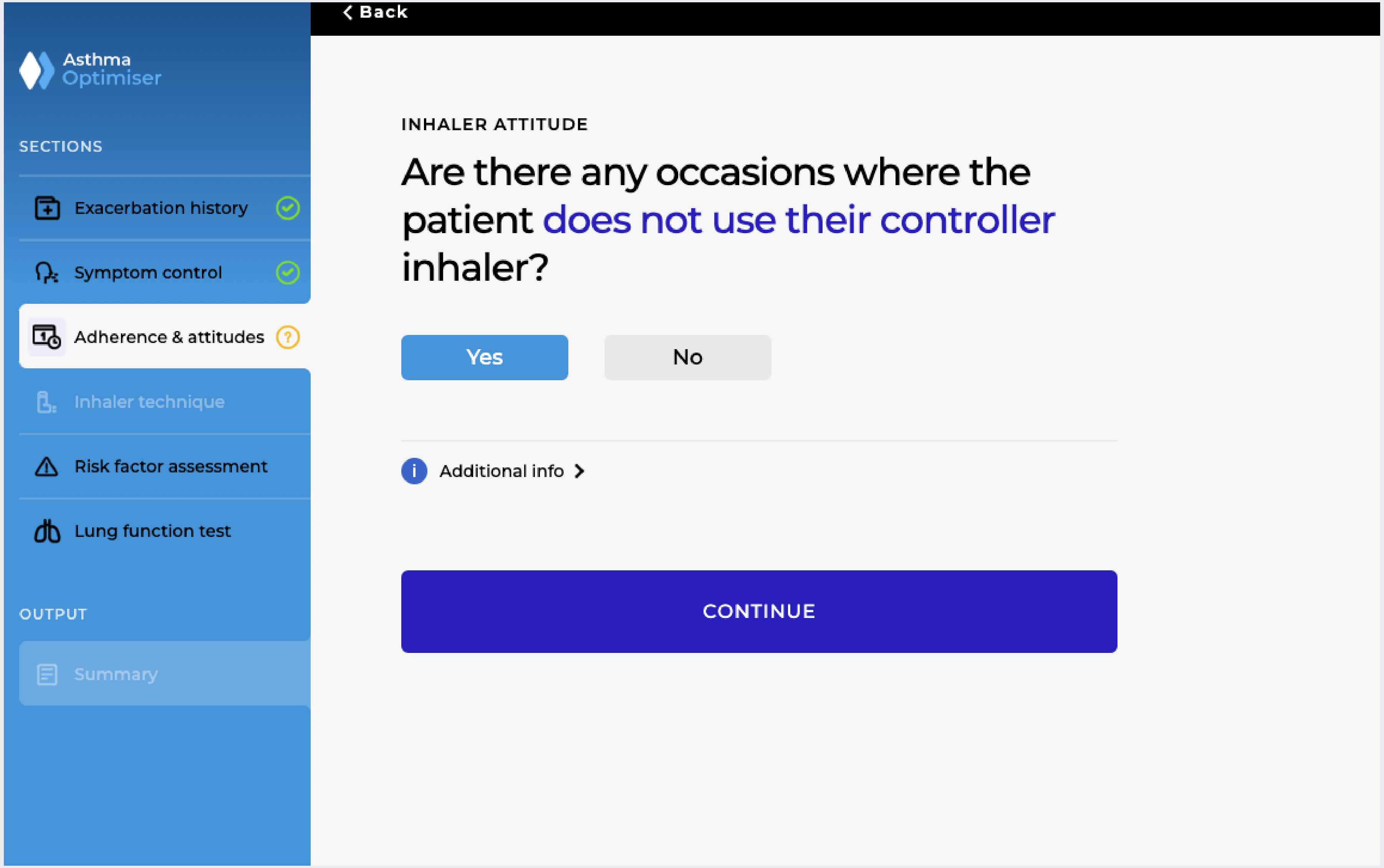
Back
Asthma Optimiser
SECTIONS
Exacerbation history
Symptom control
Adherence & attitudes
Inhaler technique
Risk factor assessment
Lung function test
OUTPUT
Summary
INHALER ATTITUDE
Are there any occasions where the patient does not use their controller inhaler?
Yes
No
Additional info
CONTINUE

16

Asthma Optimiser

SECTIONS

- Exacerbation history
- Symptom control
- Adherence & attitudes
- Inhaler technique
- Risk factor assessment
- Lung function test

OUTPUT

- Summary

Support

Terms & Conditions

Privacy Policy

Disclaimer

< Back

INHALER USAGE

## Select the reasons why your patient may not use their controller inhaler.

- [x] Forgets to use it
- [x] Unaware of need for regular usage
- [ ] Worries about side-effects
- [ ] Worries about becoming too dependent on it
- [ ] Tends to rely on the reliever inhaler for immediate relief
- [ ] Other reasons

Additional info

It is important to understand the reason why patients do not use their inhalers so that you can provide the appropriate support to help them optimise their asthma management.

CONTINUE

GP RI

17

Asthma Optimiser

SECTIONS

- Exacerbation history
- Symptom control
- Adherence & attitudes
- Inhaler technique
- Risk factor assessment
- Lung function test

OUTPUT

- Summary

Support

Terms & Conditions

Privacy Policy

Disclaimer

< Back

SKIPPING INHALER USE

## How often does the patient not take their controller, because of forgetfulness or intentionally not taking it?

- (•) Once per week
- ( ) 2-4 times/week
- ( ) >4 times/week

Additional info

It is important to understand the patient's treatment adherence in the context of control-based asthma management. Approximately 50% of adults and children on long-term therapy for asthma fail to take medications as directed at least part of the time. This might be due to forgetfulness, negligence, or both. Poor adherence might be identified by an emphatic question that acknowledges the likelihood of incomplete adherence and encourages an open discussion. Poor adherence is a potentially modifiable risk factor for future exacerbations, even in patients with few symptoms.

Please refer to the GINA 2023 report for additional information.

CONTINUE

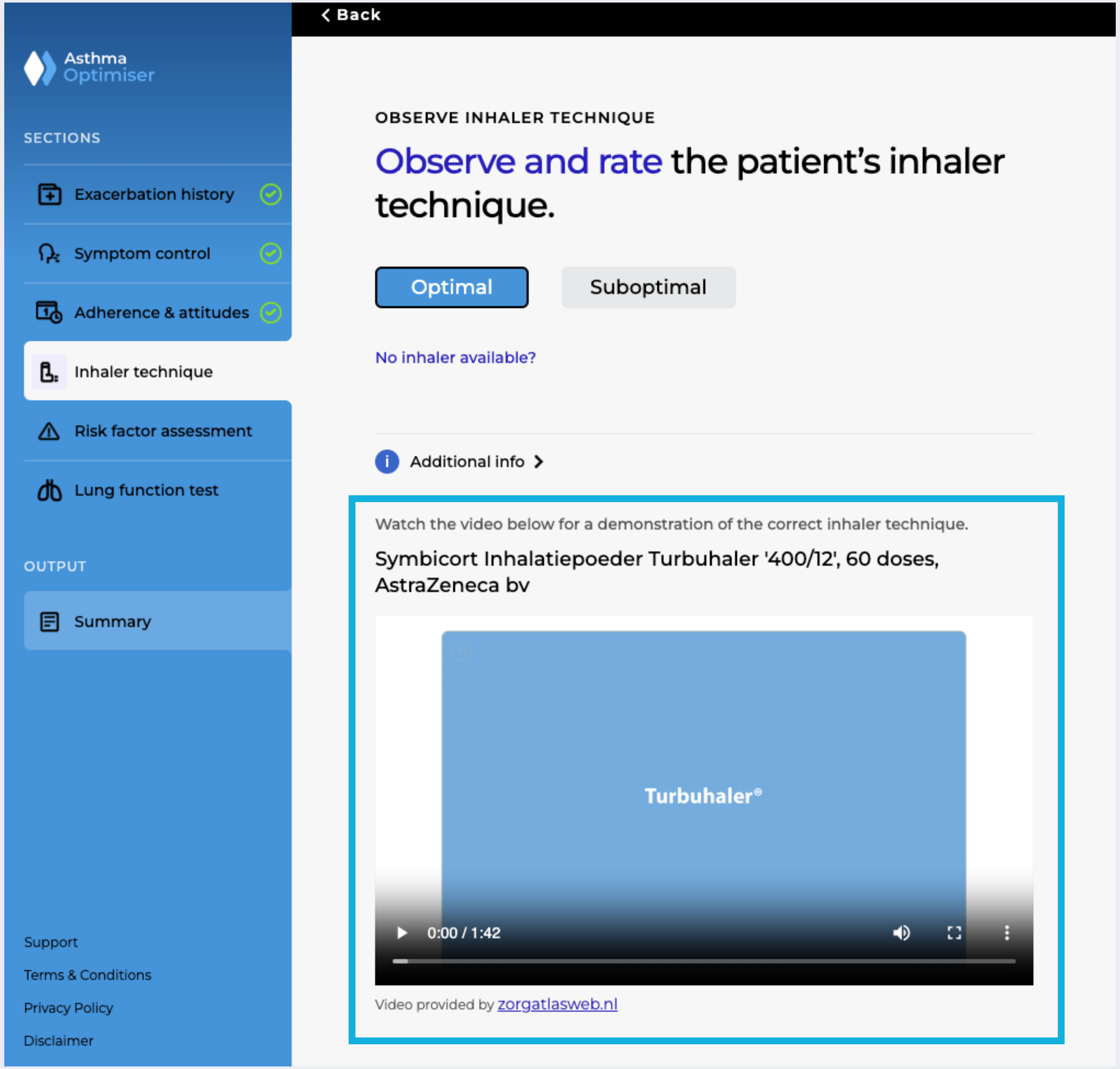

Back
Asthma Optimiser
SECTIONS
Exacerbation history
Symptom control
Adherence & attitudes
Inhaler technique
Risk factor assessment
Lung function test
OUTPUT
Summary
Support
Terms & Conditions
Privacy Policy
Disclaimer
OBSERVE INHALER TECHNIQUE
Observe and rate the patient's inhaler technique.
Optimal
Suboptimal
No inhaler available?
Additional info
Watch the video below for a demonstration of the correct inhaler technique.
Symbicort Inhalatiepoeder Turbuhaler '400/12', 60 doses, AstraZeneca bv
Turbuhaler®
0:00 / 1:42
Video provided by zorgatlasweb.nl


18

19

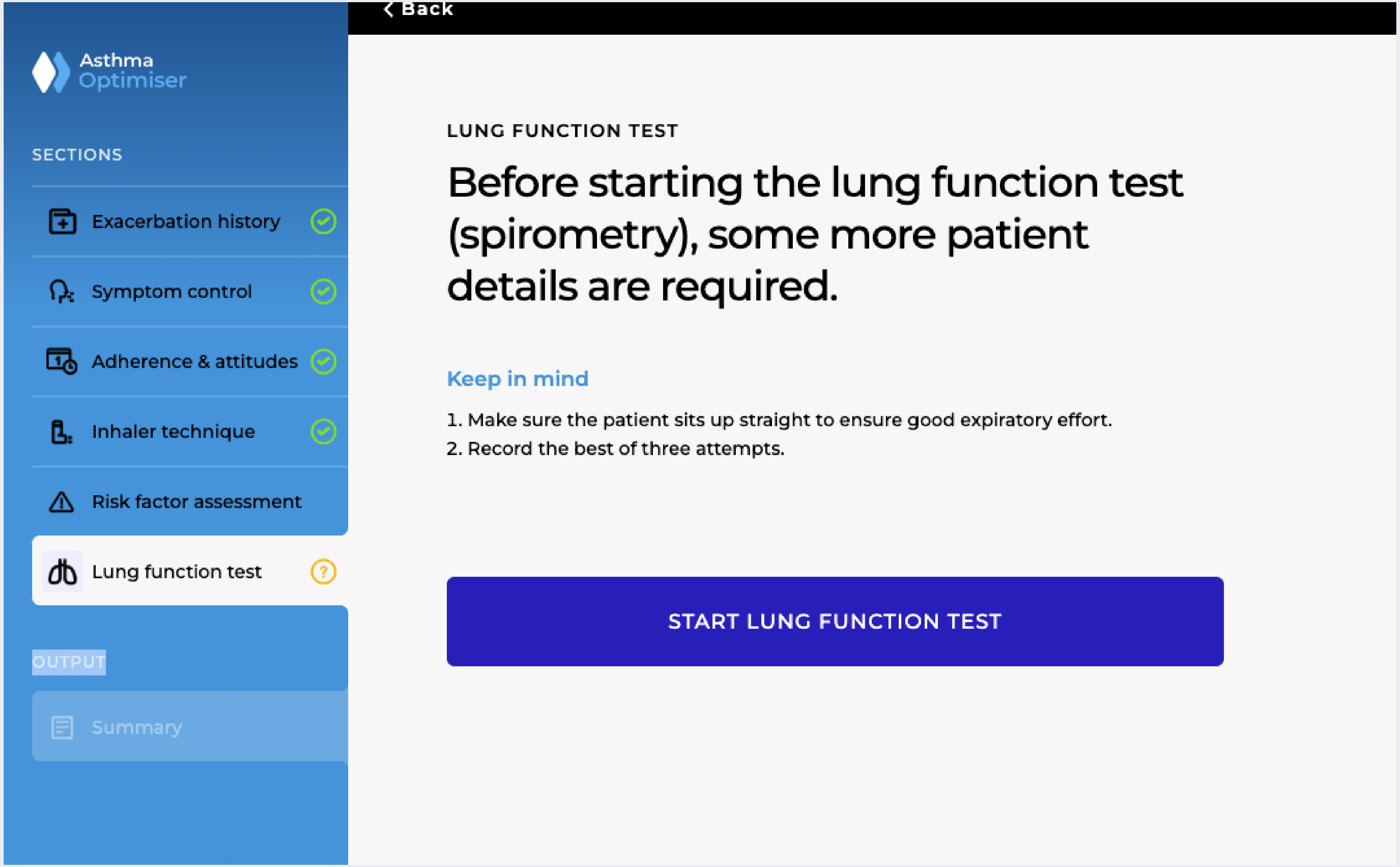

Back
Asthma Optimiser
SECTIONS
Exacerbation history
Symptom control
Adherence & attitudes
Inhaler technique
Risk factor assessment
Lung function test
OUTPUT
Summary
LUNG FUNCTION TEST
Before starting the lung function test (spirometry), some more patient details are required.
Keep in mind
1. Make sure the patient sits up straight to ensure good expiratory effort.
2. Record the best of three attempts.
START LUNG FUNCTION TEST


GPRI

20

# Lung function

- Result lung function test:
- Percentage of predicted FEV1 value = 57%

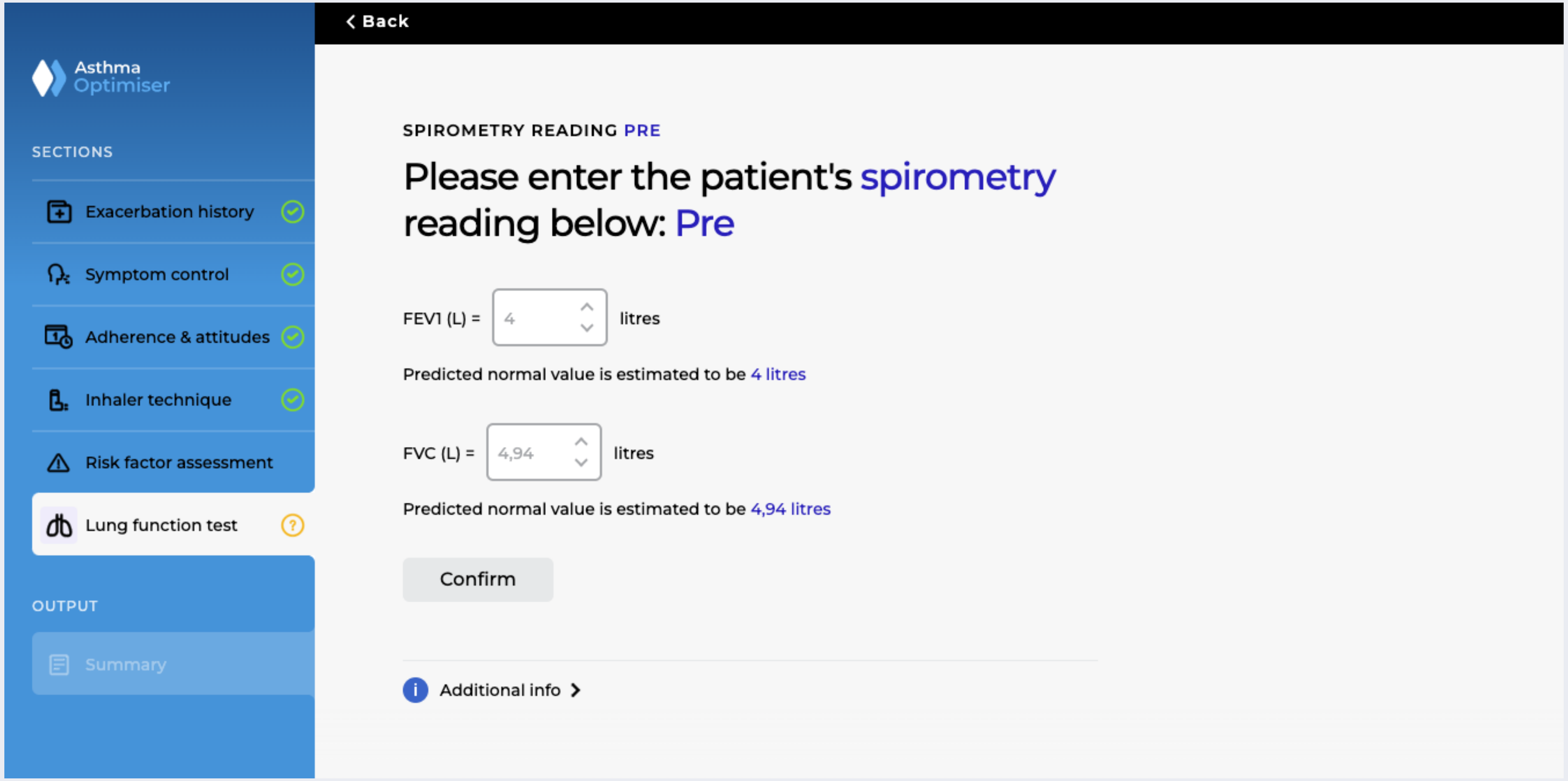

21

# Lung function

- Result lung function test:
- Percentage of predicted FEV1 value = 57%

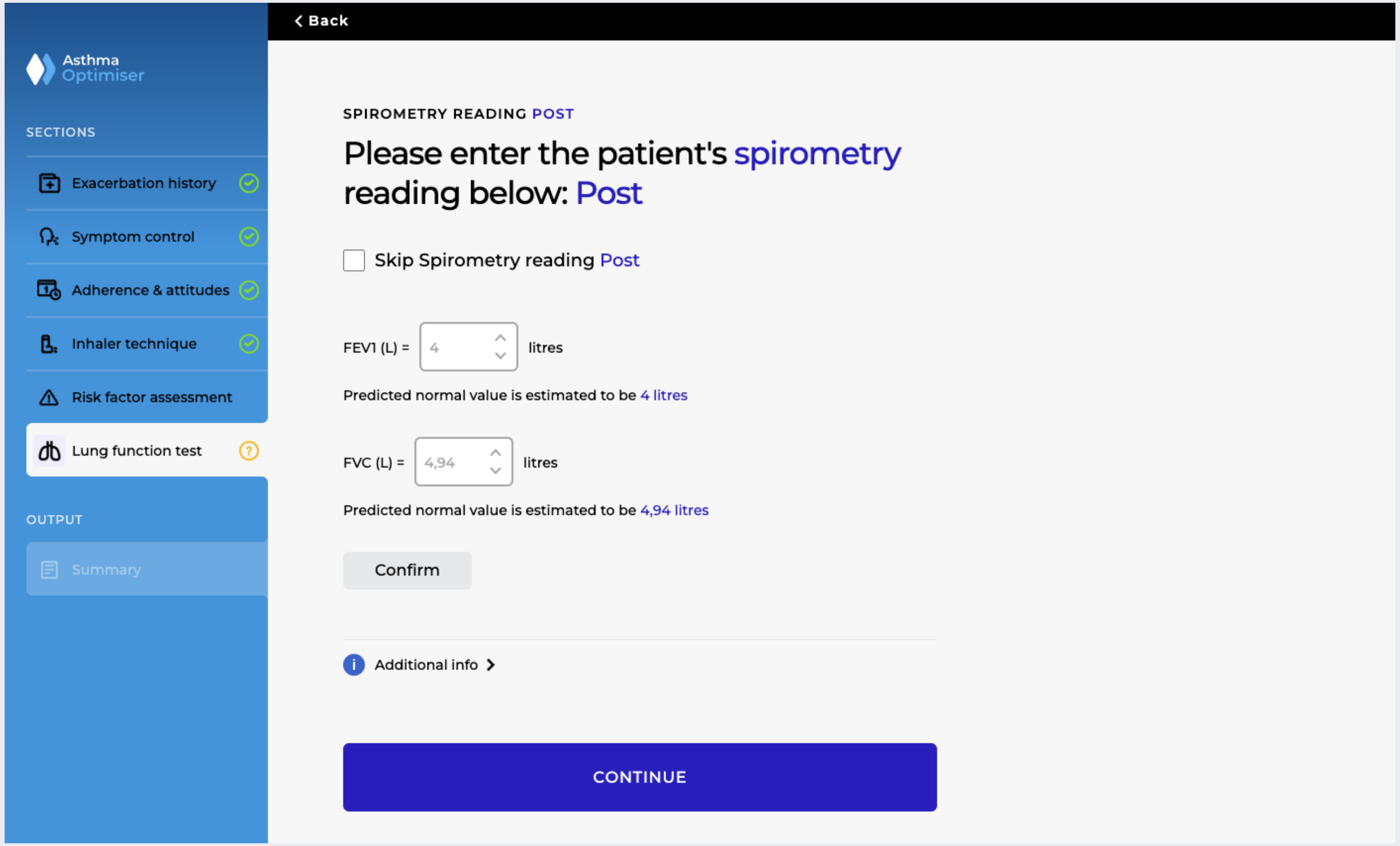

22

# Risk factor assessment

ASTHMA TRIGGERS

## Do any of these triggers make the patient's asthma worse?

- [ ] Air pollution (noxious chemicals, cleaning products)
- [ ] Anti-inflammatory drugs (e.g. aspirin)
- [ ] Respiratory viruses
- [ ] Allergies (pollen / hay fever / pets / dust / moulds / others)
- [ ] Passive smoking / vaping
- [ ] Perfume
- [ ] Other
- [ ] None

COMORBIDITIES

## Does the patient have any of these comorbidities that may contribute to their symptoms, impaired quality of life, and poor asthma control?

- [ ] Anxiety or depression
- [ ] Obstructive sleep apnoea
- [ ] Deconditioning
- [ ] Bronchiectasis
- [ ] Obesity
- [ ] Cardiac disease
- [ ] Chronic rhinosinusitis or nasal polyps
- [ ] Chronic obstructive pulmonary disease
- [ ] Gastroesophageal reflux disease
- [ ] Other
- [ ] None

SMOKING

## Is the patient smoking or did the patient smoke in the past?

- ○ Current smoker
- ○ Former smoker
- ○ Never smoker

SOCIOECONOMIC FACTORS

## Is the patient exposed to any of these socioeconomic status (SES) triggers that may contribute to their symptoms, impaired quality of life, and poor asthma control?

- [ ] Financial status
- [ ] Living conditions (mould, dust, other)
- [ ] Educational attainment
- [ ] Occupational
- [ ] Other
- [ ] No SES detected

23

# Summary

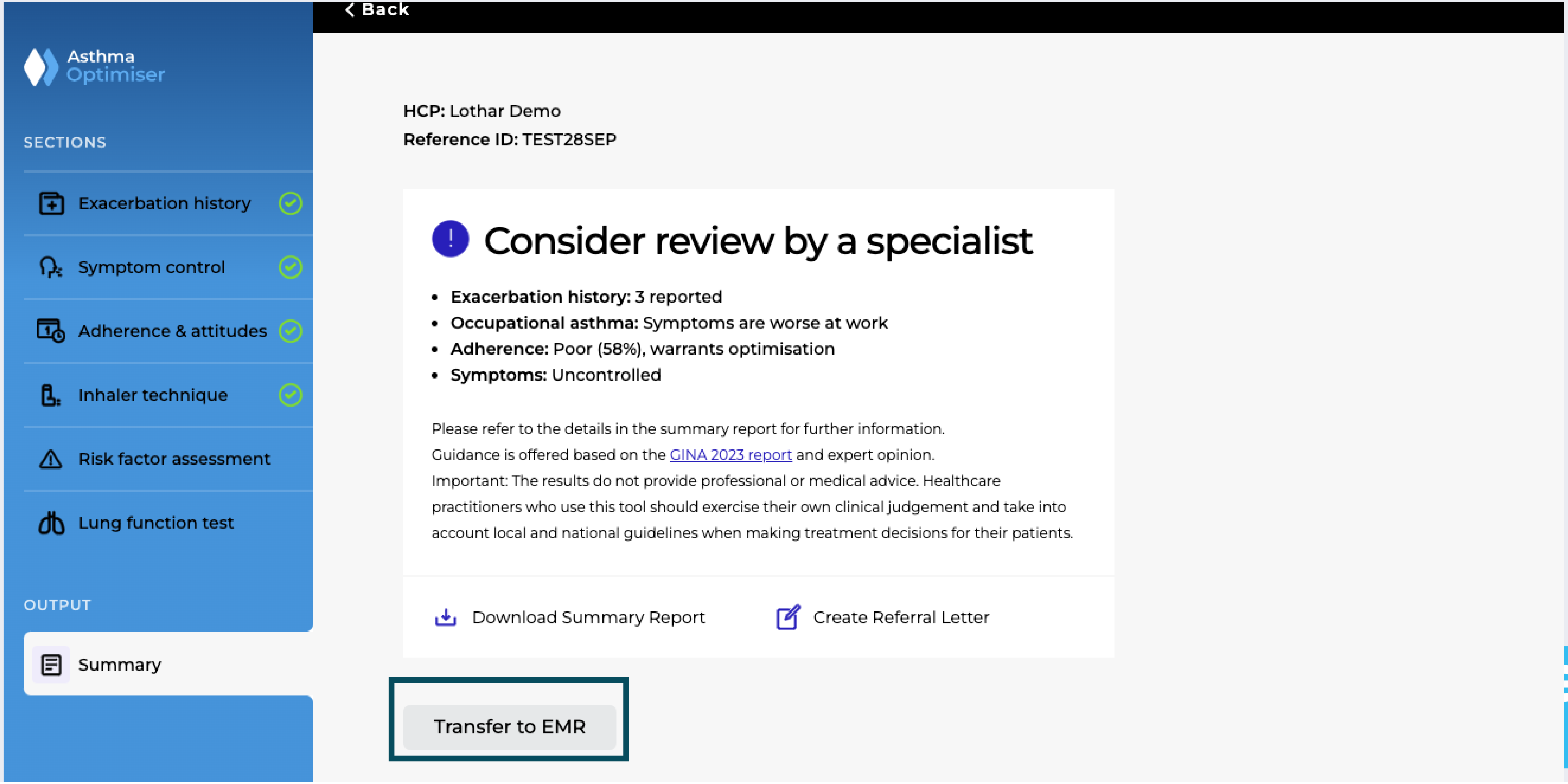

Back
Asthma Optimiser
SECTIONS
Exacerbation history
Symptom control
Adherence & attitudes
Inhaler technique
Risk factor assessment
Lung function test
OUTPUT
Summary
HCP: Lothar Demo
Reference ID: TEST28SEP
Consider review by a specialist
Exacerbation history: 3 reported
Occupational asthma: Symptoms are worse at work
Adherence: Poor (58%), warrants optimisation
Symptoms: Uncontrolled
Please refer to the details in the summary report for further information.
Guidance is offered based on the GINA 2023 report and expert opinion.
Important: The results do not provide professional or medical advice. Healthcare practitioners who use this tool should exercise their own clinical judgement and take into account local and national guidelines when making treatment decisions for their patients.
Download Summary Report
Create Referral Letter
Transfer to EMR

# 24 Summary report

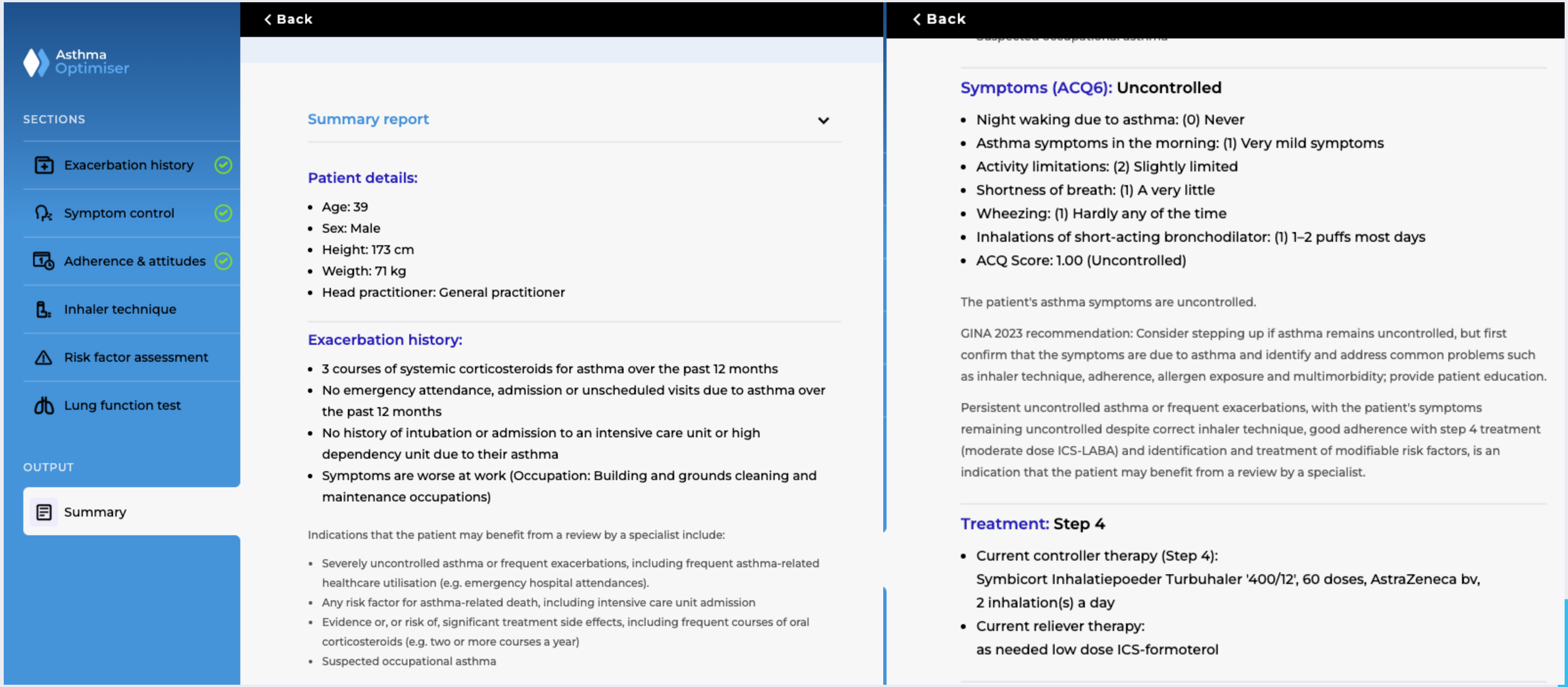


GPRI

25

# Summary report

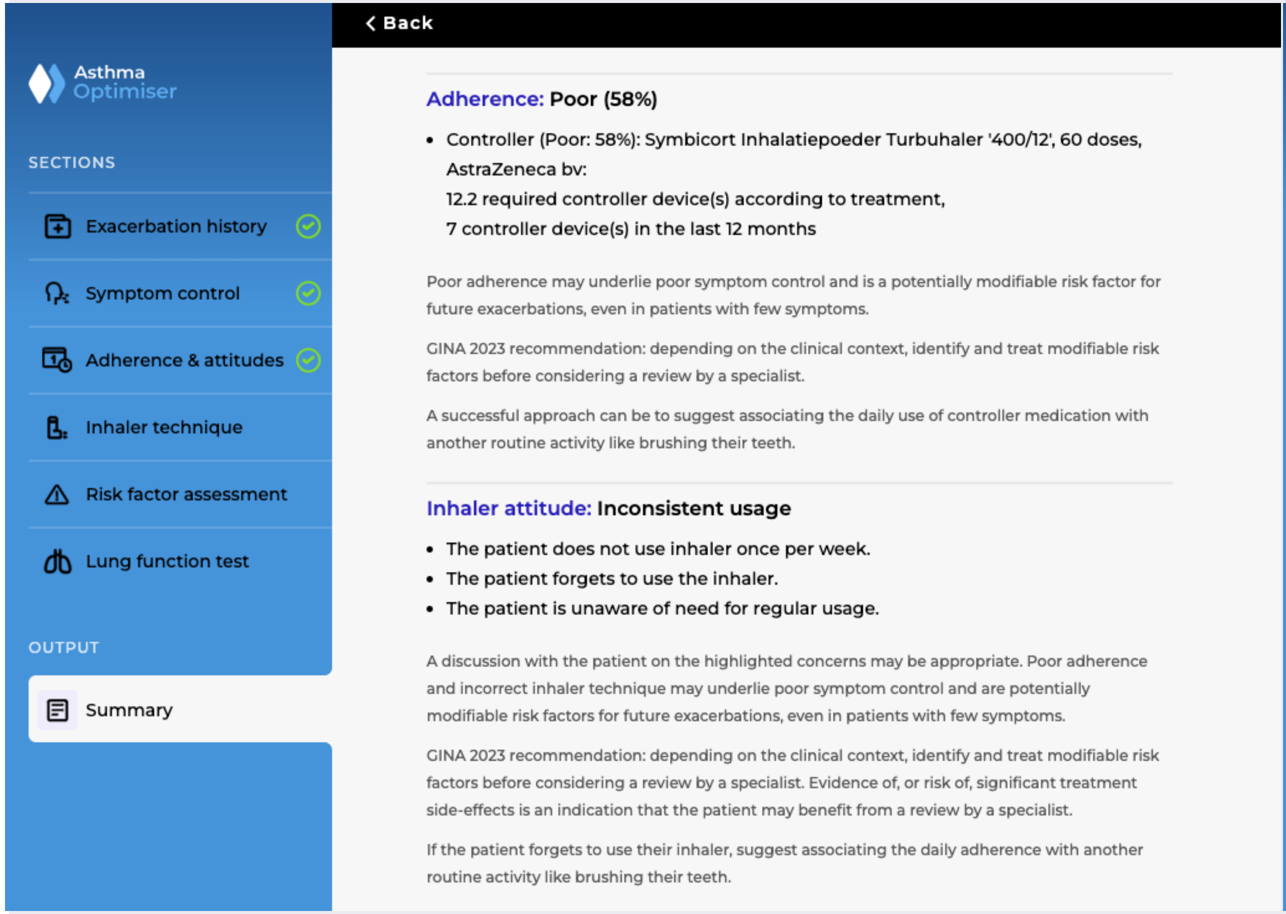


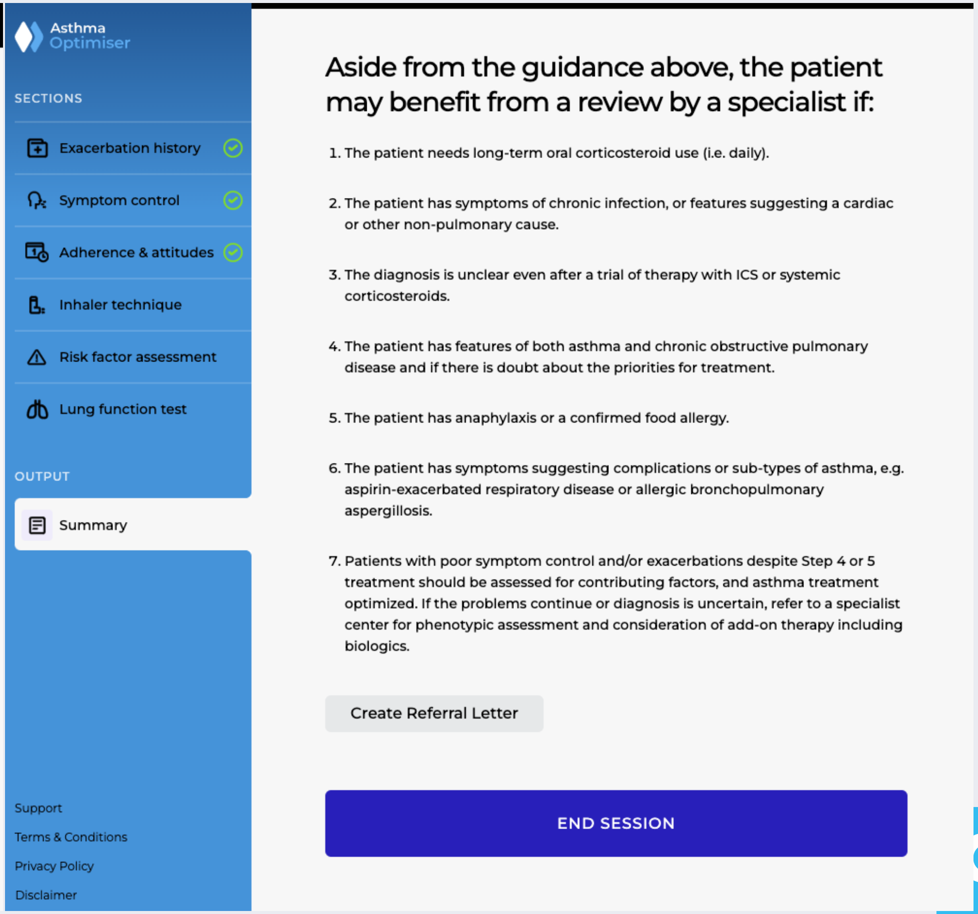

# Discussion between nurse and patient

- Patient doesn’t want to be referred to pulmonologist
- Nurse still sees opportunities to **optimise care** by improving adherence
- Medication adherence is discussed, and it is agreed that patient will return in 6 weeks for check-up

GP RI

# Consultation after 6 weeks

- Therapy adherence improved
- Despite this, ACQ-6 did not improve
- Patient is now willing to be referred to pulmonologist

GP RI

# Additional settings within AsthmaOptimiser

- Role
  - Nurse, GP, allergist, pharmacist, respiratory specialist, other specialist
- Asthma symptoms
  - GINA
  - RCP3Q: Royal college of physicians – 3 questions to assess level of asthma control in the UK
  - ACQ-6: Asthma control questionnaire – 6 questions to assess level of asthma control
- Lung function
  - Peak expiratory flow: measures the maximum speed with which a person can exhale
  - Spirometry: measures the amount and/or speed of air that can be inhaled and exhaled

GP RI
